# Antibiotic treatment for respiratory tract infections for migrants in Norway during the COVID-19 pandemic: a population-wide registry study

**DOI:** 10.1186/s12875-026-03249-x

**Published:** 2026-03-03

**Authors:** Leo Larsen, Valborg Baste, Esperanza Diaz, Guri Rortveit, Knut Erik Emberland

**Affiliations:** 1 Department of Global Public Health and Primary Care, University of Bergen, Bergen, Norway; 2 National Centre for Emergency Primary Health Care, NORCE Norwegian Research Centre, Bergen, Norway; 3 Norwegian Institute of Public Health, Oslo, Norway

**Keywords:** Primary care, respiratory tract infections, migrant health, COVID-19, epidemiology

## Abstract

**Background:**

Respiratory tract infections (RTIs) are a common reason for seeking primary health care services and are one of the main reasons for antibiotic prescriptions in primary care. The COVID-19 pandemic impacted migrants differently from the majority population in Norway.

**Objectives:**

To investigate the likelihood of antibiotic treatment for RTIs in primary care before and during the COVID-19 pandemic, and types of antibiotics used, for selected migrant populations and Norwegian-born, in Norway.

**Methods:**

Observational study on adults from Norway, Poland, Somalia, Sri Lanka, and Syria, using primary health service and demographic data from individually linked registries from Norway 2018–2021. We compared the pandemic to the pre-pandemic period using modified Poisson regression to estimate the relative risk of antibiotic treatment for RTIs during the COVID-19 pandemic.

**Results:**

The relative risk, with confidence intervals in parentheses, of antibiotic treatment for RTIs during the pandemic, by country of birth, was 0.51 (0.51–0.52) for Norway, 0.45 (0.43–0.48) for Poland, 0.41 (0.38–0.45) for Somalia, 0.45 (0.40–0.50) for Sri Lanka, and 0.54 (0.49–0.58) for Syria. Phenoxymethylpenicillin was the most common antibiotic for RTIs, accounting for 48.5–65.3% of treatments, depending on country of birth.

**Conclusion:**

All groups, irrespective of country of birth, had approximately half the antibiotic treatment rate for RTIs during the pandemic, compared to the pre-pandemic period. The most used antibiotic type was phenoxymethylpenicillin, and the distribution of antibiotic types was relatively stable over time, for all countries of birth.

## Introduction

Respiratory tract infections (RTIs) are a common reason for visit in primary care [1]. RTIs are normally self-limiting and do not require antibiotics, but when they do, the first choice for most RTIs in Norway is phenoxymethylpenicillin [2]. Norway has a stated goal of reducing antimicrobial resistance, and one of the strategies is promoting rational use of antibiotics [3]. In the initial stages of the COVID-19 pandemic, the use of antibiotics was suggested as a potential treatment for COVID-19 [4], but advised against in Norway [5]. 

The 12 March 2020, the Norwegian government announced massive interventions to prevent the spread of the COVID-19 pandemic, including restrictions on both national and international travel [6]. 

Migrants to Norway are a large and diverse group, with some overarching time trends in reasons for migration and countries of origin. In 2004, the expansion of the European Union led to an increase in Polish labour migrants [7]. Of note for our study, there was a temporary increase in the number of refugees and asylum seekers from Sri Lanka in the 1970s, from Somalia in the 1990s and 2010s, and from Syria in the 2010s.

A recent study from 18 European countries found that general practitioners’ (GPs’) perceptions that a patient wants antibiotics for an RTI were associated with a fourfold increase in likelihood of antibiotic prescription [8]. In a 2021 study, southern European migrants in Norway described higher expectations of receiving pharmaceutical treatment, such as antibiotics, than what health care professionals deemed necessary [9]. In contrast, a 2016 study reported lower purchase rates for antibiotics for migrants from five countries to Spain and Norway than for the majority populations, with variation between countries of origin [10]. 

In Norway, rates of infection, hospitalisation, and mortality, for COVID-19 were higher for people with migrant backgrounds during the pandemic [11]. These differences were not fully explained by the migrants’ socioeconomic level [12, 13], although migrants with low income and crowded housing had higher rates of notified infection and hospitalisation [14]. 

Migrants in Norway faced several barriers in adhering to COVID-19 infection prevention guidelines, such as language barriers, material barriers, frequent modification of guidelines, and lack of involvement in dissemination of information [15, 16]. However, it is unknown whether antibiotic treatment for RTIs changed during the COVID-19 pandemic for migrants in Norway. Knowledge about migrants’ contact with the health care system, and the treatment they receive, is important for future development of evidence-based and equitable health care services.

### Aims

The aim of the study was to investigate the likelihood of antibiotic treatment for RTIs for selected migrant populations and Norwegian-born consulting in Norwegian primary care before and during the COVID-19 pandemic, and types of antibiotics used.

## Methods and materials

### Primary care in Norway

All residents in Norway, regardless of migrant status, are entitled to municipal primary health care services through daytime general practice (DGP) and out-of-hours (OOH) services. These services are the primary point of contact for seeking health care, and secondary health care services are normally only accessible through referral. DGP and OOH service activity is registered in the Norwegian Registry for Primary Health Care (NRPHC), using the International Classification of Primary Care version 2 (ICPC-2) for diagnostic coding.

Antibiotics require a prescription, and all dispensing of antibiotics from pharmacies to non-institutionalised patients is registered in the Norwegian Prescription Database (NorPD).

### Data sources

We linked, on the individual level, the following national registries: NRPHC, NorPD, demographic and residence data from Statistics Norway (SSB), and time of death from the Norwegian Cause of Death Registry (NCDR).

### Study population

We included adults (≥ 18 years) born in Norway, Poland, Somalia, Sri Lanka, or Syria, registered in Norway in the years 2018–2021. We included these migrant groups for their population sizes, the availability of published Norwegian primary care and pandemic research for them, and to represent a variety of migration, pandemic, and health care seeking experiences [7, 10–18]. 

As the data set provided by SSB did not contain updated data on newborn, immigrated, or emigrated individuals for 2021, we used data from 2020 as a proxy for 2021 for education and residence data. Person-years were estimated from individual residence data and time of death.

### Definitions and variables

We defined an RTI consultation as having a reimbursement code for a consultation (in-person or electronic) in DGP or OOH services, with a diagnosis code from our ICPC-2 RTI definition (Supplementary Table 1), based on previous work [19]. 

We defined an RTI episode as an index RTI consultation and any subsequent RTI consultations within 30 days of the previous RTI consultation, extending the period to 30 days after each included RTI consultation. There was no upper limit for RTI episode duration or number of RTI consultations per RTI episode. Patient characteristics for the RTI episode were defined by the index RTI consultation.

We included data on standard (non-reimbursed) prescription dispensing of all oral systemic antibiotics in Anatomical Therapeutic Chemical (ATC) classification system subgroup ‘antibacterial drugs’ (J01). For oral systemic antibiotics with more than one ATC code (vancomycin (A07AA09) and metronidazole (P01AB01)), we included both codes. Antibiotics used exclusively for urinary tract infections were not included (pivmecillinam, mecillinam, trimethoprim, nitrofurantoin, and methenamine). We grouped antibiotics into phenoxymethylpenicillin (J01CE01), other penicillins (other J01C), tetracyclines (J01A), macrolides (J01FA), and other antibiotics.

We defined an ‘RTI consultation with antibiotic treatment’ as an RTI consultation followed by an antibiotic dispensing within seven days. We defined an ‘RTI episode with antibiotic treatment’ as having at least one RTI consultation with antibiotic treatment. There was no upper limit for number of antibiotic treatments per RTI consultation or RTI episode, and each dispensing was counted separately for analyses on antibiotic types.

The main outcome was RTI episode*s* with antibiotic treatment. The exposure was the COVID-19 pandemic period, as compared to the pre-pandemic period. To avoid possible seasonal bias, we defined the pre-pandemic period as 12 March 2018 to 30 November 2019 and the pandemic period as 12 March 2020 to 30 November 2021.

For country of birth, we included patients born in Poland, Somalia, Sri Lanka, and Syria – and patients born in Norway to Norwegian-born parents, as predefined by SSB [20]. To avoid diluting the effect of having a migrant background in analyses, we did not include individuals born in Norway with any foreign-born parents, or foreign-born with any Norwegian-born parents. We used length of stay in Norway to account for integration of migrants over time.

We divided age into three groups: 18–41, 42–66, and ≥ 67 years, the standard age of retirement in Norway. Binary sex categories were used as predefined by SSB.

We used the categories of educational level as predefined by SSB: ‘unknown or no education’, ‘below upper secondary education’, ‘upper secondary education’, ‘tertiary vocational education’, ‘higher education, short’ (up to four years), and ‘higher education, long’ (more than four years) [21]. Missing values were included in ‘unknown or no education’.

RTI episodes grouped by service type, as ‘daytime general practice’, ‘out-of-hours’, or ‘mixed’, depending on where the consultation(s) took place.

### Statistical analysis

All data cleaning, analysis, and visualisation was done using Stata/SE version 18.5 (StataCorp LLC).

For each country of birth, we provided number of RTI episodes and rate (RTI episodes per 1000 person-years), by patient and episode characteristics. Length of stay was presented as mean and standard deviation (SD). We visualised monthly RTI episodes and antibiotic treatment and the distribution of antibiotic treatment groups for RTIs using rates.

We estimated the relative risk (RR) of RTI episodes having at least one antibiotic treatment in the pandemic period, compared to the pre-pandemic period, using modified Poisson regression, as a log-binomial model failed to converge [22]. The analysis was stratified by country of birth, and adjusted for sex, age, education, and length of stay in Norway for migrants. Both crude and adjusted estimates were reported with 95% confidence interval (CI).

## Results

The study population comprised 3 721 856 adults, including 95.6% (*n* = 3 557 870) born in Norway, 2.9% (*n* = 104 015) from Poland, 0.7% (*n* = 26 604) from Somalia, 0.7% (*n* = 24 019) from Syria, and 0.3% (*n* = 9 348) from Sri Lanka. Mean length of stay in Norway for migrant patients in RTI episodes varied considerably between patients from Sri Lanka (25.1 years, SD 9.2), Somalia (14.9 years, SD 7.5), Poland (10.9 years, SD 7.0), and Syria (5.7 years, SD 4.7).

Over the whole study period, patients from Sri Lanka had the highest RTI episode rate (308 RTI episodes per 1000 person-years), and patients from Poland the lowest (180 RTI episodes per 1000 person-years) (Table 1). All migrant groups had higher RTI episode rates in 2021 compared to 2020, while for Norwegian-born, the number decreased slightly.

**Table 1:** Respiratory tract infection (RTI) episodes and rate, by episode characteristics and country of birth (Norway 2018–2021)

	Country of birth									
	Norway		Poland		Somalia		Sri Lanka		Syria	
	Number of RTI episodes	RTI episode rate per 1000 person-years	Number of RTI episodes	RTI episode rate per 1000 person-years	Number of RTI episodes	RTI episode rate per 1000 person-years	Number of RTI episodes	RTI episode rate per 1000 person-years	Number of RTI episodes	RTI episode rate per 1000 person-years
Total	2,670,456	203	66,724	180	24,117	245	11,099	308	22,265	258
Calendar year										
2018	701,694	215	14,624	162	5553	230	2976	332	4598	234
2019	678,417	207	14,347	156	5552	227	2749	306	5405	255
2020	662,664	202	16,334	175	6236	252	2486	276	5360	241
2021	627,681	191	21,419	226	6776	269	2888	320	6902	296
Age										
18–41	1,187,444	257	47,786	221	17,802	265	3641	333	17,576	270
42–66	1,009,226	186	18,173	122	5954	205	6912	307	4516	224
67+	473,786	154	765	127	361	149	546	214	173	173
Sex										
Female	1,582,698	240	35,305	268	13,883	299	5909	337	9303	285
Male	1,087,758	167	31,419	132	10,234	197	5190	281	12,962	242
Education										
None registered	3937	210	452	218	1708	234	144	254	1628	260
Basic school	626,915	227	9790	197	13,691	244	3777	294	10,360	282
Upper secondary	1,066,428	201	22,479	159	4609	259	3730	347	1753	273
Tertiary vocational	85,837	188	704	195	164	291	316	305	154	253
Higher (≤ 4 years)	683,646	206	10,877	242	2119	261	2022	333	2727	234
Higher (> 4 years)	199,027	163	10,497	241	281	173	600	243	754	204
Service type										
DGP	2,269,645	173	58,565	158	19,964	203	9720	270	18,740	217
OOH	271,908	21	5190	14	2740	28	786	22	2429	28
Mixed	128,903	10	2969	8	1413	14	593	16	1096	13

*DGP* Daytime general practice, *OOH* Out-of-hours

The rates of RTI episodes decreased with increasing age, except among patients from Poland (Table 1). The RTI episode rates were higher for female than male patients for all countries of birth.

**Fig. 1:**
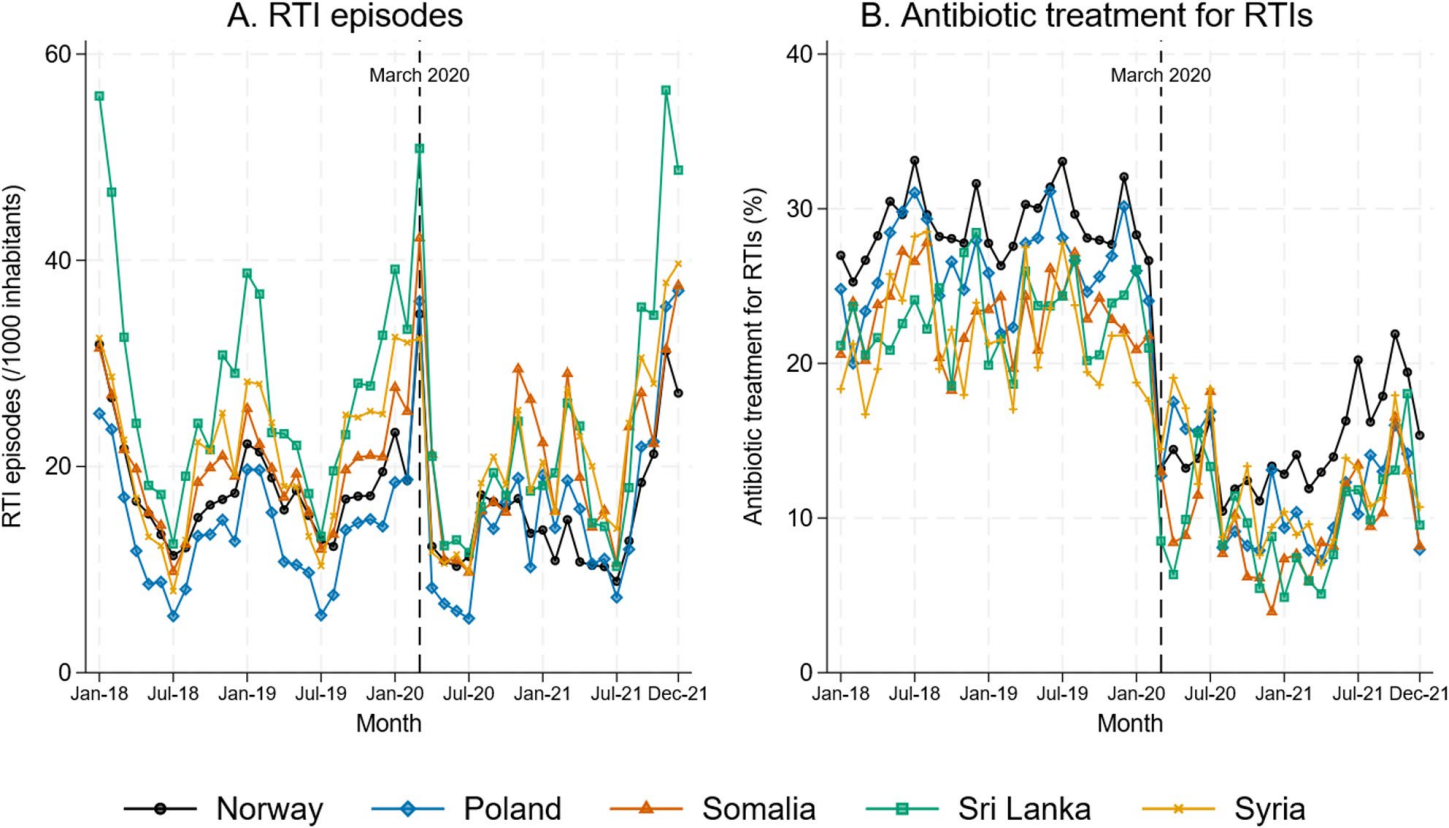
Monthly respiratory tract infection episodes (RTIs) (**A**), and antibiotic treatment for RTIs (**B**), by country of birth (Norway 2018–2021)

All countries of birth displayed seasonal variation in monthly RTI episodes rates with higher rates in the winter, and a drop in both RTI episodes and RTI antibiotic treatment rate in the months following March 2020 (Fig. 1).

Overall, patients born in Norway had the highest rate of antibiotic treatment for RTIs during both pre-pandemic (29.1%) and pandemic (14.7%) periods (Table 2). When comparing the pandemic to the pre-pandemic period, there was a large reduction in antibiotic treatment rate for RTIs for all countries of origin. The largest reduction was found for patients from Somalia (RR 0.41, CI 0.38–0.45) and the smallest for patients from Syria (RR 0.54, CI 0.49–0.58) and Norway (RR 0.51, CI 0.51–0.52). There were only small differences in RR between the crude and adjusted models.

**Table 2:** Likelihood of antibiotic treatment for respiratory tract infections in Norway during COVID-19 pandemic, compared to pre-pandemic period

	Pre-pandemic (12 March 2018–30 November 2019)		Pandemic (12 March 2020–30 November 2021)		Relative risk			
	No. of episodes	% with antibiotic	No. of episodes	% with antibiotic	Crude RR	95% CI	Adjusted* RR	95% CI
Country of birth								
Norway	1,098,345	29.1	1,032,923	14.7	0.50	0.50–0.51	0.51	0.51–0.52
Poland	22,701	26.0	29,850	11.3	0.43	0.42–0.45	0.45	0.43–0.48
Somalia	8970	23.3	10,457	9.4	0.40	0.37–0.43	0.41	0.38–0.45
Sri Lanka	4398	22.9	4143	10.1	0.44	0.40–0.50	0.45	0.40–0.50
Syria	8157	21.6	9569	11.6	0.54	0.50–0.58	0.54	0.49–0.58

*Adjusted for sex, age, education, and length of stay in Norway

*RR* Relative risk, *CI* Confidence interval

**Fig. 2:**
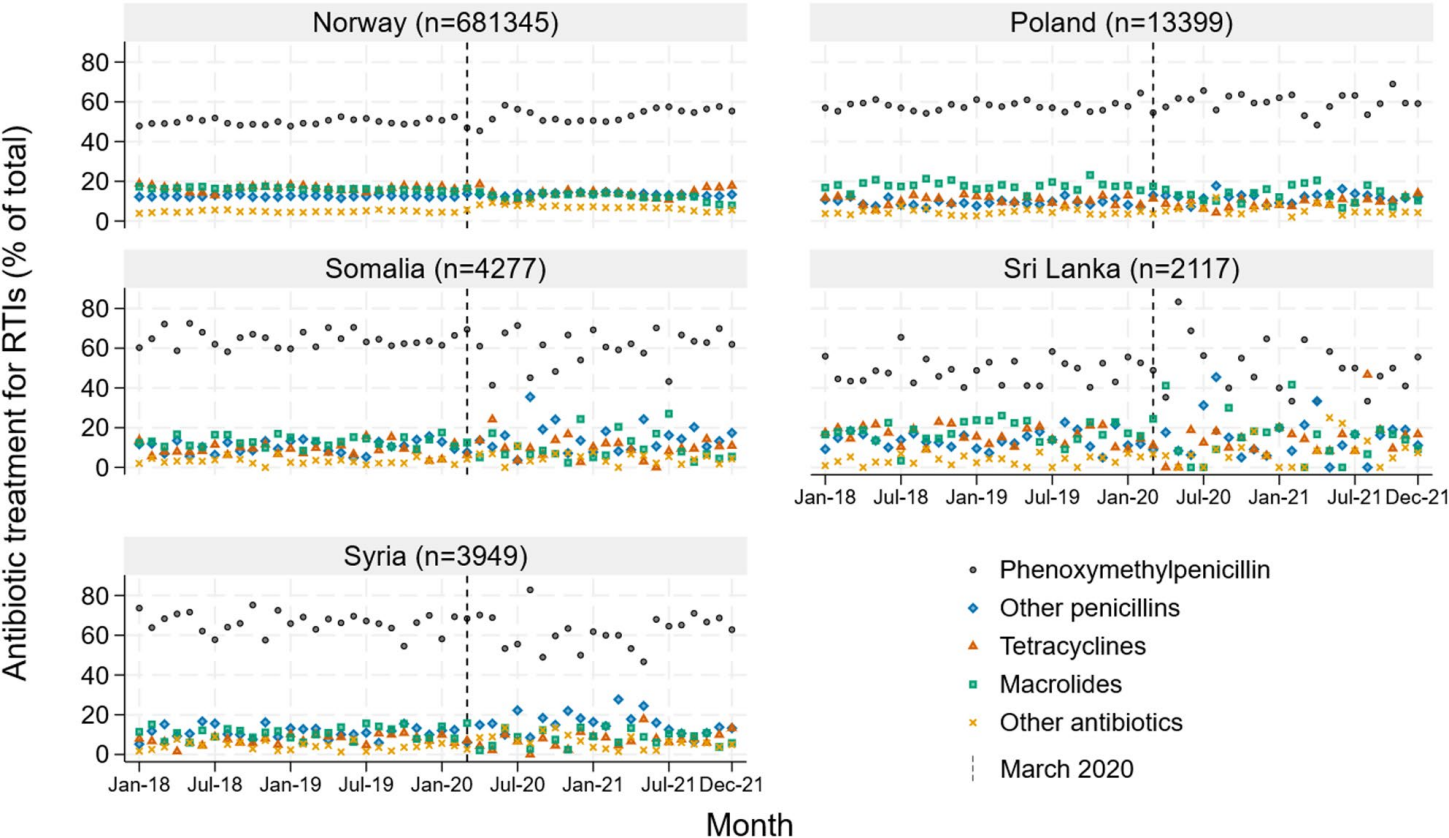
Distribution of antibiotic treatment for respiratory tract infections (RTIs) by country of birth (Norway 2018–2021)

There was no clear change in the distribution of antibiotic types after March 2020, for any of the observed countries of birth (Fig. 2). Overall, phenoxymethylpenicillin was the most common antibiotic type for RTI episodes among patients from Syria (65.3%), Somalia (63.9%), Poland (58.5%), Norway (50.9%), and Sri Lanka (48.5%).

## Discussion

Over the study period, patients born in Somalia, Syria, and Sri Lanka had a higher primary care RTI episode rate than patients born in Norway, while patients born in Poland had a lower rate. All migrant patient groups received lower rates of antibiotics for RTIs than patients born in Norway, both before and during the COVID-19-pandemic. For all countries of birth, the use of antibiotics for RTIs was halved during the pandemic compared to before the pandemic. Phenoxymethylpenicillin was the most frequently used antibiotic type for RTIs for all populations both before and during the pandemic, with some differences in frequency between countries of birth.

Migrants from Poland, Syria, and Somalia had higher rates of hospitalisation for COVID-19 than Norwegian-born, even when adjusting for socioeconomic factors [12]. This aligns with our finding of higher RTI episode rates for those migrant groups in 2021 than for Norwegian-born, but do not explain the lower rates in 2020 for migrants from Poland.

A previous study on use of OOH services among migrants in Norway showed that patients born in Poland had fewer consultations for RTIs in OOH compared to patients born in Norway, but that patients born in Somalia had more [18]. These findings correspond to ours. An explanation of lower health care usage among migrants from Poland could be due to the healthy migrant effect, especially since labour is a common reason for migration from Poland. This notion is strengthened by findings from a registry study from Poland, which estimated the pre-pandemic yearly incidence rate for upper RTI episodes to be 508 per 1000 inhabitants per year [23]. 

Migrants born in Sri Lanka have shown high COVID-19 vaccination rate as of 2021, similar to Norwegian-born, while migrants born in Somalia and Poland showed lower rates [17]. The same distribution was not found for RTI episode rates, and shows that RTI health care seeking is not a uniform issue.

Migrants born in Syria, a group with a large proportion of refugees, have reported lower rates of antibiotic use in Norway than in Syria [24]. In our study, migrants born in Syria had a higher RTI episode rate than Norwegian-born, for all years, age groups, sexes, educational levels, and service types. They also had the lowest rate of antibiotic treatment for RTIs in the pre-pandemic period. In a 2020 paper, Harris et al. reported a perception among some Norwegian GP’s that refugees preferred their problems solved using medication [25], similar to findings from a Dutch study reporting that GPs’ felt recently arrived immigrants were more likely to expect antibiotics than Dutch natives [26]. Considering that GPs’ perceptions of patient expectations affect prescribing [8], it would be reasonable to expect that migrants have higher rates of antibiotic treatment for RTIs. However, this is in contrast with our results, which are supported by previous studies showing that migrants receive less antibiotic treatment than the majority populations [10]. Studies investigating the reasons for this discrepancy are needed.

A major strength of this study is the scope and completeness of the data. We analysed data from all DGP and OOH service consultations and pharmacy dispensing of antibiotics in Norway over a 4-year period. The prospective registration of the data in the registries is a methodological strength. The use of the pandemic period as exposure in the study does have some advantages, as it was a societal change affecting everyone. However, this means that it is difficult to identify specific reasons for the observed changes. The study only obtained information on dispensed antibiotics in Norway. Antibiotics prescribed outside of Norway or self-medication for patients with other access, such as leftover medication from previous prescriptions, would not have been captured. Lastly, the observational design of the study limits the ability to draw conclusions regarding causality.

The linking of all antibiotic prescriptions to RTI consultations may have overestimated the rate of antibiotic treatment, as some prescriptions may have been for other reasons. However, as RTIs are the most common infections and reason for antibiotic prescription, we do not believe this substantially reduces the validity of the study. Furthermore, if there was an overestimation, we have no reason to believe that this was differentiated between periods or countries of origin. The RTI episode definition with 30-day follow-up was likely better suited for capturing lower RTIs and could have underestimated the number of episodes for upper RTIs due to their shorter length.

A weakness of the study is that we did not have data on the socioeconomic situation of the study participants and therefore could not fully adjust for socioeconomic differences, even when adjusting for education. This limits the extent to which the migrant groups can be compared to one another. For the main outcome, estimates will not have been affected by differences in socioeconomic level between countries of birth, since these were stratified.

Travel restrictions during the pandemic could potentially have impacted the ability of migrants to travel for health care. This could have led to more health care seeking behaviour in Norway during the pandemic. However, we consider it unlikely that migrants frequently travel to their birth country for treatment of RTIs, as these conditions are typically self-limiting and short. A study from Tromsø showed that only 3.7% of travellers in a general Norwegian population purchased antibiotics abroad but reported an association between growing up abroad and purchasing prescription antibiotics abroad [27]. We lack information on the extent of self-medication in our study, but it is possible that travel restrictions during the pandemic impacted the likelihood of travelling to Norway with medication obtained abroad. This could have led to an underestimation of the decrease in likelihood of antibiotic treatment for RTIs among migrants during the pandemic.

Findings from this study are potentially relevant for countries with similar primary health care systems, but translating findings to other communities and migrant populations should be done with care and subject matter knowledge to avoid overgeneralisation.

## Conclusion

There was a reduction in the antibiotic treatment rate for RTIs during the pandemic for patients from all studied countries of birth. For all countries of birth in the study, the distribution in antibiotic types used for RTIs was largely unchanged over the study period.

## Data Availability

The data that support the findings of this study were owned by the registries (Norwegian Registry for Primary Health Care, Norwegian Prescription Database, Statistics Norway, Norwegian Cause of Death Registry), with restrictions on availability of data, which were used under license for the current study, and so are not publicly available. Due to changes in the administration of Norwegian health registries, requests to access data should be directed to Helsedata (https://helsedata.no).

## Abbreviations

ATC: Anatomical Therapeutical Chemical
CI: Confidence interval
DGP: Daytime general practice
GP: General practitioner
ICPC: International Classification of Primary Care
ICPC: 2–International Classification of Primary Care version 2
NCDR: Norwegian Cause of Death Registry
NorPD: Norwegian Prescription Database
NRPHC: Norwegian Registry for Primary Health Care
OOH: Out–of–hours
RR: Relative risk
RTI: Respiratory tract infection
SD: Standard deviation
SSB: Statistics Norway
